# Timing of puberty and school performance: A population-based study

**DOI:** 10.3389/fendo.2022.936005

**Published:** 2022-08-05

**Authors:** Maria Suutela, Päivi J. Miettinen, Silja Kosola, Ossi Rahkonen, Tero Varimo, Annika Tarkkanen, Matti Hero, Taneli Raivio

**Affiliations:** ^1^ New Children’s Hospital, Helsinki University Hospital, Pediatric Research Center, Helsinki, Finland; ^2^ Faculty of Medicine, University of Helsinki, Helsinki, Finland; ^3^ Stem Cells and Metabolism Research Program, Research Programs Unit, Faculty of Medicine, University of Helsinki, Helsinki, Finland; ^4^ Department of Public Health, Faculty of Medicine, University of Helsinki, Helsinki, Finland; ^5^ Department of Physiology, Faculty of Medicine, University of Helsinki, Helsinki, Finland

**Keywords:** adolescence, puberty, school health, academic achievement, school performance, age at peak height velocity

## Abstract

**Objective:**

To determine whether the timing of puberty associates with school performance.

**Methods:**

Growth data on 13,183 children born between 1997 and 2002, were collected from child health clinics and school healthcare and school performance data from school records. Age at peak height velocity (PHV) marked pubertal timing. The relationships between age at PHV and average grades in mathematics, native language, English, and physical education from school years 6 (end of elementary school; age 11-12 years), 7 (start of middle school; 12-13 years), and 9 (end of middle school; 14-15 years) were modeled using generalized estimating equations and linear mixed models, adjusted for the month of birth and annual income and education levels in school catchment areas.

**Results:**

The mean (SD) age at PHV was 13.54 (1.17) years in boys and 11.43 (1.18) years in girls. In girls, age at PHV was associated with grades in mathematics (β=0.041–0.062, p<0.005) and physical education (β=0.077–0.107, p<0.001) across the study years, and in school year 9, also with grades in English (β=-0.047, 95%CI -0.072 to -0.021, p<0.001). Among boys, only the grades in physical education were related to age at PHV across the study years (β=0.026–0.073, p<0.01) and in middle school the grades in mathematics decreased dramatically.

**Conclusions:**

In both sexes, the timing of puberty was associated with the grades in physical education, and in girls, with academic achievement. The decrease in boys’ mathematics grades and sex difference in academic achievement were unexplained by the timing of puberty.

## Introduction

Functional organization of the brain during adolescence follows nonlinear puberty-dependent trajectories (1). Brain imaging studies imply a relationship between puberty and decreasing density of grey matter in cortical and subcortical areas (2–4), increasing white matter density (3–6), and an increase in the whole-brain functional network (7). Puberty-related maturation of the hippocampus, amygdala, and cortical gray matter differs between girls and boys (8), and, in girls, white matter matures earlier than in boys even after adjusting for puberty stages (9). The role of sex steroids in brain maturation is unclear. For instance, Paus *et al.* have shown that bioavailable testosterone levels in boys correlated with whole brain and white matter volume (4), and, conversely, testosterone deficiency before adulthood has been attributed to diminished spatial ability (10). Although adolescence and puberty exert sex-specific effects on the brain, the changes do not seem to be binary regardless of the temporo-spatial expression of androgen- and estrogen-receptors in the various areas of the brain (11). Since the 19^th^ century, a rapid secular trend towards earlier timing of puberty has been reported among girls (12) and this trend seems to continue in the late 20^th^ and early 21^st^ century though at a slower pace (13). In boys, a secular trend in pubertal timing has recently been reported in Sweden; the age at peak height velocity (PHV) was 1.2 months earlier for every decade increase in birth year after controlling for BMI (14).

Whether differences in pubertal timing are connected to academic achievement is still unclear. The relationship between school performance and timing of puberty has been probed already in the 1960s, when Douglas and Ross investigated the relationship between the timing of sexual maturation and school performance in 5000 children born in the first week of 1946 in the UK (15). They found an unequivocal positive relationship between early puberty and better school performance in both boys and girls. In 1991, Dubas et al. reported on school performance and puberty timing among 252 adolescents from a Midwestern city (16). In their study, pubertal timing effects were apparent during the early adolescent years on grades in social studies, language arts, and literature; interestingly, late-developing boys received the lowest grades, and late-developing girls received the highest (16). The results from the above studies show a relationship between extremes of puberty timing and school performance. Nevertheless, these results can hardly be extrapolated to the 21st century given the secular trends in puberty timing, changes in the numerous factors known to affect pubertal timing (13, 14, 17), and changes in education. Additionally, cultural differences and the relatively small sample size of the Midwestern study may affect the generalizability of the findings. In 2021, Torvik et al. studied the relationship between the average grade across all subjects at age 16 and puberty timing of 13,477 British children. They found that earlier puberty was associated with higher academic achievement both in boys and girls but that early age at menarche in girls was associated with lower academic achievement (18).

For children and adolescents, one of the most important social determinants of health is education (19). To this end, Finland has consistently been among the best countries in the OECD’s Programme for International Student Assessment (PISA), where the skills of 15-year-olds are measured in reading, mathematics, and science (20). Unfortunately, according to the PISA results from 2018, the gap in school performance between Finnish boys and girls is one of the widest in the world. This gap was the highest in reading, but girls outperformed boys also in mathematics and science (21). In all countries that participated PISA in 2018, girls outperformed boys in reading but in mathematics and science there was variation by country (21). Similar results have been reported in prior PISA studies (21, 22). Reasons for this gap remain unresolved, yet a plausible reason is the difference in the timing of puberty between boys and girls.

In this study, the aim was to investigate the relationship between puberty and school performance in a population-based setting among 13,183 adolescents in the second largest city in Finland. The primary hypothesis was that the timing of puberty is one factor explaining variation in academic subjects and physical education in both sexes.

## Subjects and methods

### Finnish education and health monitoring system

In Finland, comprehensive education begins the calendar year during which children turn seven and continues for nine years (school years 1-9). Typically, students attend elementary school (school years 1-6) and transfer to middle school (school years 7-9) at the age of 12-13 years. After middle school, students apply to either academic high school or vocational education. In early childhood, families visit child health clinics where a minimum of 15 health checks are conducted prior to school age. More than 99% of Finnish children participate in this follow-up (23). When a child starts elementary school, preventive health and wellbeing services continue within schools. In general, a preventive care nurse meets every student annually to follow growth and development as well as other health and wellbeing measures. In school years 1, 5, and 8, a physician participates in an extensive health check (24, 25). Children attend school health care also in high school or vocational education although the measurement frequency is not as regular as during lower school years.

### Study population, collection of growth data

**Figure 1:**
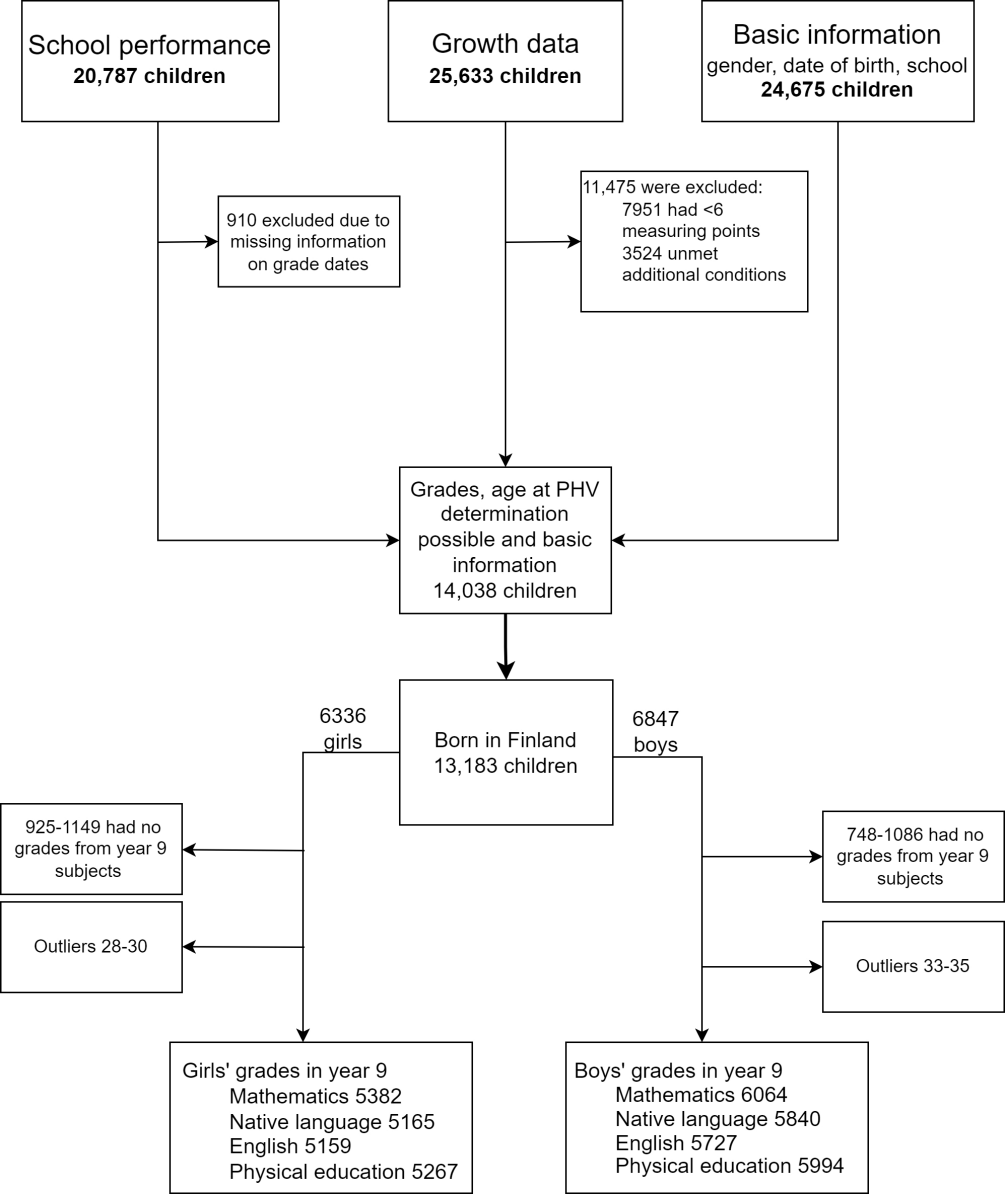
Study outline. School grades of children born between 1997 and 2002 were gathered from comprehensive schools in Espoo and integrated with growth and background data.

We collected information on children born between 1997 and 2002 who attended a comprehensive school in the city of Espoo, the second largest city in Finland, with approximately 293,000 inhabitants (end of 2020) (26). Since the study is entirely register based, no ethical permission was required according to the Finnish Medical Research Act. The Helsinki University Hospital and the city of Espoo approved the study. The children attended 94 different comprehensive schools, 36 of which provided education in school years 7-9. The children’s growth and health data from the visits to child health clinics and school health services were obtained from the integrated patient information systems (Effica^®^, Tieto Inc.). Demographic data included sex and date of birth. Growth data were available for 25,633 children, school grades for 20,787 children, and basic information (sex, date of birth, schools attended) for 24,675 children. Children born outside of Finland were excluded because insufficient language skills could have affected school performance in academic subjects, the reason for birth abroad was unknown (adoption, etc.), and some early growth data were missing. Our final study population included 13,183 children (6847 boys and 6336 girls) (**Figure 1**). The growth data included all height measurements from school health care. Unfortunately, data on final heights were not available.

### Assessment of pubertal timing

Determination of the age at PHV is explained in detail in the Supplementary Materials. In many previous studies, visually defined peak height velocity has been used as a marker for puberty (27–29) but this is an inefficient way in large study groups. In a study by Aksglaede et al., the height velocity was defined as the difference in heights at two adjacent measurements divided by the difference in time between these measurements (30). This value was assigned at the age in the middle of this period. Age at peak height velocity was the maximal height velocity. There are also mathematical models for estimating peak height velocity (31, 32). In the study by Simpkin et al., four different methods were compared and applied to a real cohort of 3123 boys. There were significant differences in the mean estimated ages at PHV (12.70 years-14.26 years) and the Pearson’s correlation coefficients to assess the level of agreement between the methods varied between 0.0734-0.8105 (33). For instance, in the SITAR-method the age at PHV bias was small but some convergence issues arose, and the utility of this method was limited when the measurement frequency was sparse or the measurement error large (33). Because there was no widely approved method in determining the age at PHV, we chose to develop our own and validate it in the study group by comparing it to visually defined ages at PHV. In our data, the final height was not known and the frequency of measurements in school health care was limited. For these and subsequent statistical analyses, Python programming language (Python 3.7.4) was used along with the following packages/libraries: NumPy, Matplotlib, Pandas, Scikit-learn, Scipy, Seaborn, and Statsmodels. For linear mixed modelling, R programming language (4.1.1) was used with the lme4 package. After validation of the method, 7^th^ degree polynomial fit was employed to each child’s growth data, and thereafter the function was derived to identify the age at PHV (see eMethods and **eTable1** in the Supplement for details). 13,285 children had sufficient data for the timing of puberty and were born in Finland.

#### Socio-economic status

Socio-economic status was included at the residential area level because most children attend a comprehensive school within their residential area. Measures of socio-economic status in the area included the average annual income level (median) and the education level (percentage of residents with a degree from tertiary education) (34).

#### Grades on academic achievement and physical education

Grades on academic achievement and physical education were obtained from an integrated electronic school monitoring system Wilma^®^ (StarSoft Inc., part of Visma Enterprise Inc) used in all schools in Espoo. Wilma^®^ includes a register for school grades and is also a communication platform between school and home. The Wilma registry includes information on subject codes, subject grades and dates when given, along with basic information such as school name (usually in years 6 and 9).

Mathematics, native language (Finnish, Swedish or other native language studied at school), and English were chosen as measures of academic achievement. English is the most popular first foreign language studied in Finnish schools (35). In contrast to PISA, science grades were incompatible since the subjects change during comprehensive education. While younger children participate in environmental studies, children in middle school study biology, geography, physics, chemistry, and health education as separate subjects. To counter the academic subjects, physical education was selected as another subject possibly affected by pubertal development. Numerical grades were available at least from school years 5 to 9. In the Finnish school system, grades vary from 4 (failed) to 10 (excellent). We were particularly interested in grades from school year 9 (starting age 14-15 years) because they determine the educational path afterwards, and this is also the year of the PISA assessment. An in-depth analysis of the associations of grades from school years 6 and 7 with the timing of puberty was conducted for children whose grades from year 9 were available. Worth noting is that some children had grades only from some school years due to moving to or from Espoo during their comprehensive education.

### Comparison of academic achievement and the age at PHV

Different models were used to compare school performance with age at PHV. Girls and boys were analyzed separately due to differences in pubertal timing. To see the general trend between age at PHV and school grades, LOWESS (Locally Weighted Scatterplot Smoothing) was used. In Python, Statsmodels module provides a lowess function. Grades in mathematics, native language, English, and physical education were compared separately with pubertal timing. Ages at PHV that deviated more than 3 SDS from the mean (SDS < -3 or SDS > 3) were omitted as outliers to minimize measurement error. Generalized estimating equations (GEE) and a linear mixed model were then fitted to these cases along with linear regression which led to similar results as the preceding. In GEE models, age at PHV and month of birth were explanatory variables. GEE model estimates population average effects and schools were analyzed as clusters. The covariance was exchangeable meaning that all observations over time have the same correlation. If less than 10 children attended a school, these children had to be omitted from both the GEE and linear mixed model analyses because schools were used as clusters.

In the linear mixed model, age at PHV and month of birth were child-level fixed effects and income level and education level in the district were residential area-level fixed effects. The residential area and the school within the residential area were chosen as random effects. If the residential area failed to explain the variance, only the school was chosen as a random effect. Equation for the linear mixed model is


y=Xβ+Zu+ϵ

where y is the outcome variable, X is a design matrix of the fixed effects (age at PHV and month of birth are child-level fixed effects and income level and education level are residential area-level fixed effects), β is column vector of the fixed-effects regression coefficients, Z is the design matrix for the random effects (the residential area and the school within the residential area) and u is the vector of the random effects and ϵ is the residuals not explained by the model. **eTable2** in the Supplement summarizes the multilevel structure of the data. The model was constructed stepwise, and the final model was compared with a null model with no fixed effects using maximum likelihood (**eTable3** in the Supplement). Akaike Information Criterion was used as a measure of model fit. The final results were fitted using restricted maximum likelihood. P-values for parameter estimates were calculated using Satterthwaite’s approximation for degrees of freedom. Intraclass correlation coefficients and variance inflation factors for each level were counted. The variance inflation factors were less than five in all cases which indicates no significant multicollinearity in the model. P-value <0.01 was accepted to indicate statistical significance due to multiple testing.

Of approximately 12,000 children for whom the definition of age at PHV was not possible, 7462 had no basic information (the school attended, etc.) or grades from school years 6, 7, or 9. These children most likely lived in the city of Espoo only in early childhood (before school year 6) or during high school years. Therefore, no descriptive information about these children is available and they were excluded from the analyses. The study population included the children who attended school years 6,7 or 9 in the city of Espoo and therefore the majority of the excluded children did not fulfil this criterion. Furthermore, for 3968 children, the age at PHV could not be defined, but they had basic information and school grades from school years 6, 7, or 9 in mathematics. The number varies slightly depending on the subject and year level. **eTable4** in the Supplement compares the grades and socio-economic status of children included in the analyses to those who had school grades from year 9 and the school attended was known but who lacked age at PHV. Different socio-economic classes are well represented in both the excluded and included study groups. One possible reason for the difference in the results between the included and excluded ones is that some of the excluded children were not born in Finland, since in previous studies, immigrants are known to have worse school performance in Finland (21, 36). Some of the excluded children have also attended small hospital or special schools. Thus, the exclusion of children due to unavailable data or immigration does not likely affect the generalizability of our findings.

## Results

### Distribution of age at PHV

**Figure 2:**
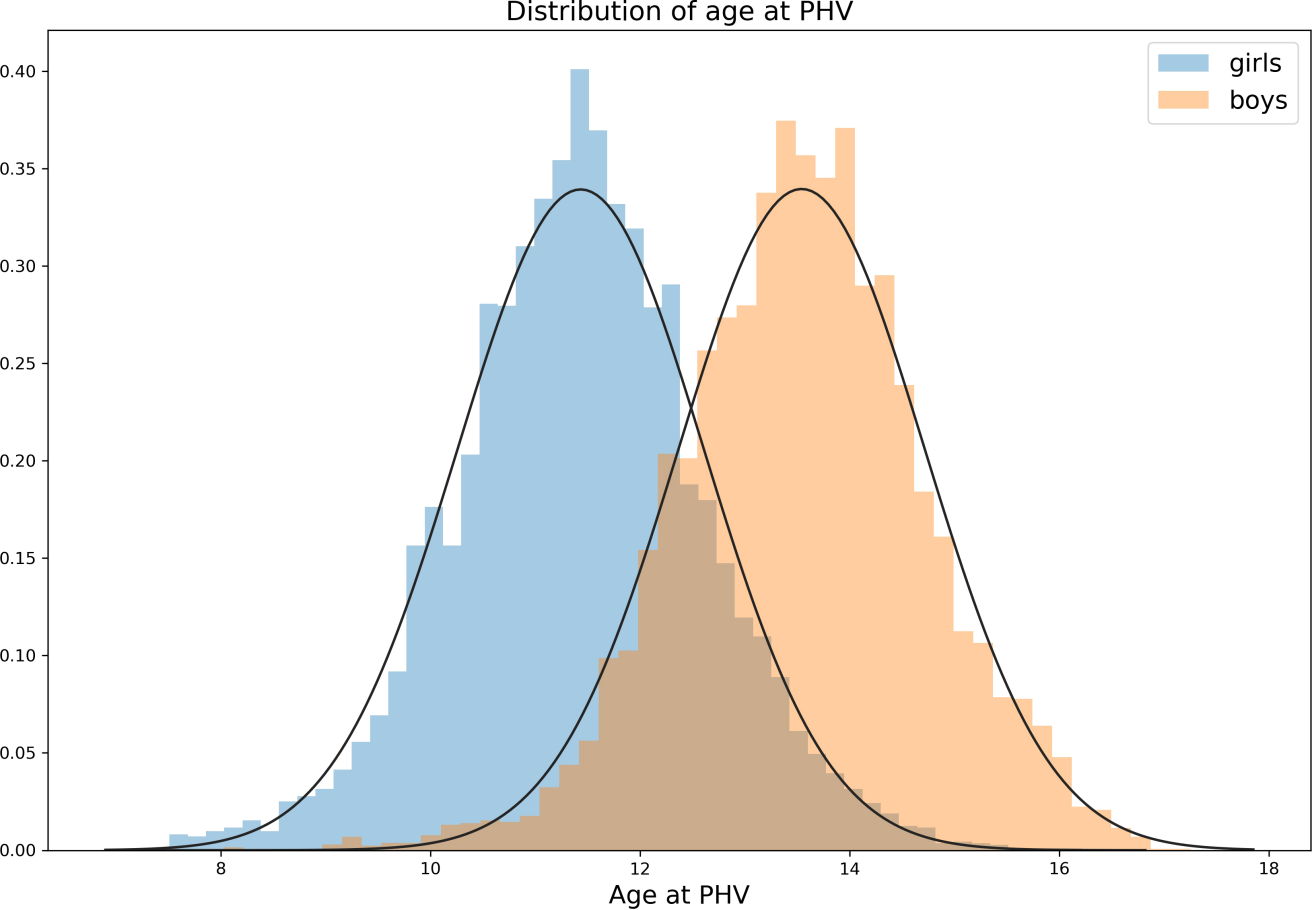
Distribution of age at PHV determined with 7th degree polynomial function for girls (n=6392) and boys (n=6893). The mean age at PHV for girls was 11.43 years ( ± 1.18 SD) and for boys 13.54 years ( ± 1.17 SD).

The average age at PHV was 13.54 years ( ± 1.17 SD) for boys and 11.43 years ( ± 1.18 SD) for girls. The normal distribution of the results is presented in **Figure 2** and Supplementary **eTable5** summarizes the characteristics of the data. On average, the children had 13.31 ( ± 3.72 SD) measurements and the mean age at the last measurement was 16.59 years ( ± 1.24SD) years.

### Relationship between age at PHV and school performance

The relationship between age at PHV, mathematics, native language, English, and physical education was investigated by GEE and linear mixed models (**Table 1**). In girls, the average grades in mathematics in years 6, 7, and 9 and age at PHV correlated positively in both models with coefficients between 0.0407 (95% CI 0.014 to 0.068, p=0.003) and 0.0619 (95% CI 0.037 to 0.087, p<0.001) and the correlation persisted even once the girls with the age at PHV <-2 SDS and >+2 SDS were excluded (data not shown). In year 9 late maturing girls (age at PHV +2 SDS) were thus estimated to score 0.20 grade points (effect size 0.19) higher in mathematics than early maturing girls (age at PHV -2 SDS). In boys, age at PHV showed no association with the average grade in mathematics. In native language, age at PHV showed no association with average grades in any school year except in year 6 in boys (**Table 1**). The correlation between age at PHV and the average grade in English language in year 9 in girls was, unlike in the other examined measures of school performance, negative in both models (**Table 1**). This translates to a 0.22 grade point difference (effect size 0.24) in favor of early maturing girls (age at PHV -2SDS) compared to late maturing girls (age at PHV +2SDS). The correlation in English remained also after exclusion of early and late maturing children (age at PHV <-2 SDS or >2SDS). In the other school years, no association was found. In boys, no correlation between age at PHV and average grade in English was evident. Age at PHV exhibited a positive association with physical education in both sexes (**Table 1**) in school years 6, 7, and 9. In year 9 late maturing girls (age at PHV +2 SDS) were estimated to score 0.50 grade points (effect size 0.61) higher in physical education than early maturing girls (age at PHV -2 SDS) and late maturing boys 0.12 grade points higher (effect size 0.06) (GEE-model) than early maturing boys.

**Table 1:** Results from GEE and linear mixed models for boys and girls.

		Mathematics			Native language			English			Physical education		
		n	Coef for age at PHV (95% CI)	p-value	n	Coef for age at PHV(95% CI)	p-value	n	Coef for age at PHV(95% CI)	p-value	n	Coef for age at PHV(95% CI)	p-value
**Girls**	**Year 9**												
	GEE- model*	5366	**0.0440 (0.016, 0.071)**	**0.002**	5152	0.0058 (-0.017 0.029)	0.62	5141	**-0.0458(-0.069,-0.022)**	**<0.001**	5252	**0.1065 (0.087, 0.126)**	**<0.001**
	Linear mixed model**		**0.0433 (0.015, 0.072)**	**0.003**		0.0053 (-0.017, 0.027)	0.63		**-0.0469(-0.072,-0.021)**	**<0.001**		**0.1058 (0.084, 0.128)**	**<0.001**
	**Year 7**												
	GEE- model*	5241	**0.0619 (0.037, 0.087)**	**<0.001**	4997	0.0237 (0.004, 0.043)	0.02	4937	-0.0194 (-0.047, 0.008)	0.17	5107	**0.0952 (0.075, 0.116)**	**<0.001**
	Linear mixed model**		**0.0614 (0.035, 0.088)**	**<0.001**		0.0236 (0.004, 0.043)	0.02		-0.0196 (-0.045, 0.006)	0.13		**0.0949 (0.077, 0.113)**	**<0.001**
	**Year 6**												
	GEE- model*	4540	**0.0422 (0.014, 0.071)**	**0.004**	4338	0.0057 (-0.017, 0.028)	0.62	4392	-0.0284(-0.054,-0.003)	0.03	4441	**0.0772 (0.052, 0.102)**	**<0.001**
	Linear mixed model**		**0.0407 (0.014, 0.068)**	**0.003**		0.0050 (-0.016, 0.026)	0.64		-0.0293(-0.056,-0.003)	0.03		**0.0771 (0.059, 0.096)**	<**0.001**
**Boys**	**Year 9**												
	GEE- model*	6055	0.0005 (-0.025,0.026)	0.97	5807	0.0020 (-0.022, 0.027)	0.87	5716	-0.0274(-0.054,-0.001)	0.05	5985	**0.0262 (0.008, 0.044)**	**0.005**
	Linear mixed model**		-0.0003(-0.030,0.029)	0.99		0.0018 (-0.022, 0.025)	0.88		-0.0281 (-0.054,-0.003)	0.03		0.0258 (0.005, 0.047)	0.01
	**Year 7**												
	GEE- model*	5917	0.0194(-0.008,0.047)	0.16	5673	0.0190 (-0.006, 0.044)	0.13	5462	-0.0094 (-0.035, 0.016)	0.47	5826	**0.0324 (0.008, 0.057)**	**0.01**
	Linear mixed model**		0.0190(-0.007,0.045)	0.16		0.0188 (-0.002, 0.040)	0.08		-0.0097 (-0.035, 0.015)	0.44		**0.0320 (0.014, 0.050)**	**<0.001**
	**Year 6**												
	GEE- model*	5102	0.0305(0.006, 0.055)	0.02	4894	**0.0352 (0.012, 0.059)**	**0.003**	4856	0.0089 (-0.018, 0.035)	0.51	5040	**0.0733 (0.053, 0.094)**	**<0.001**
	Linear mixed model**		0.0297 (0.003, 0.056)	0.03		**0.0344 (0.012, 0.057)**	**0.002**		0.0082 (-0.017, 0.034)	0.53		**0.0729 (0.054, 0.092)**	**<0.001**

* In GEE- model, age at PHV and month of birth as explanatory variables and schools as clusters.

** In linear mixed model, age at PHV, month of birth, income level and percentage with tertiary level degree as fixed effects and residential area and school within residential area as random effects (fitted with REML).

Age at PHV is compared with average grades in mathematics, native language, English and physical education in school years 6, 7, and 9.

The values in bold indicate that they are statistically significant.

In linear mixed models, the month of birth correlated negatively with the average grades in all subjects in school years 6 and 7 except for English in year 7 (**eTable6** in the Supplement). In year 9, the month of birth exhibited an inverse association only with the grade in physical education in boys (β=-0.0172, 95%CI -0.024 to -0.010, p<0.001) (**eTable6**). The income and education level of the residential area showed no association with school performance in years 7 and 9. In year 6, the education level of the residential area correlated positively with boys’ average grade in mathematics (β=0.0244, 95%CI 0.013 to 0.036, p<0.001) and native language (β=0.0162, 95% CI 0.006 to 0.026, p=0.006). The residential area explained 0.0-7.7% and the school within the residential area 1.4-11.4% of the variance after the variance was explained by fixed effects.

### Sex differences in school performance

**Figure 3:**
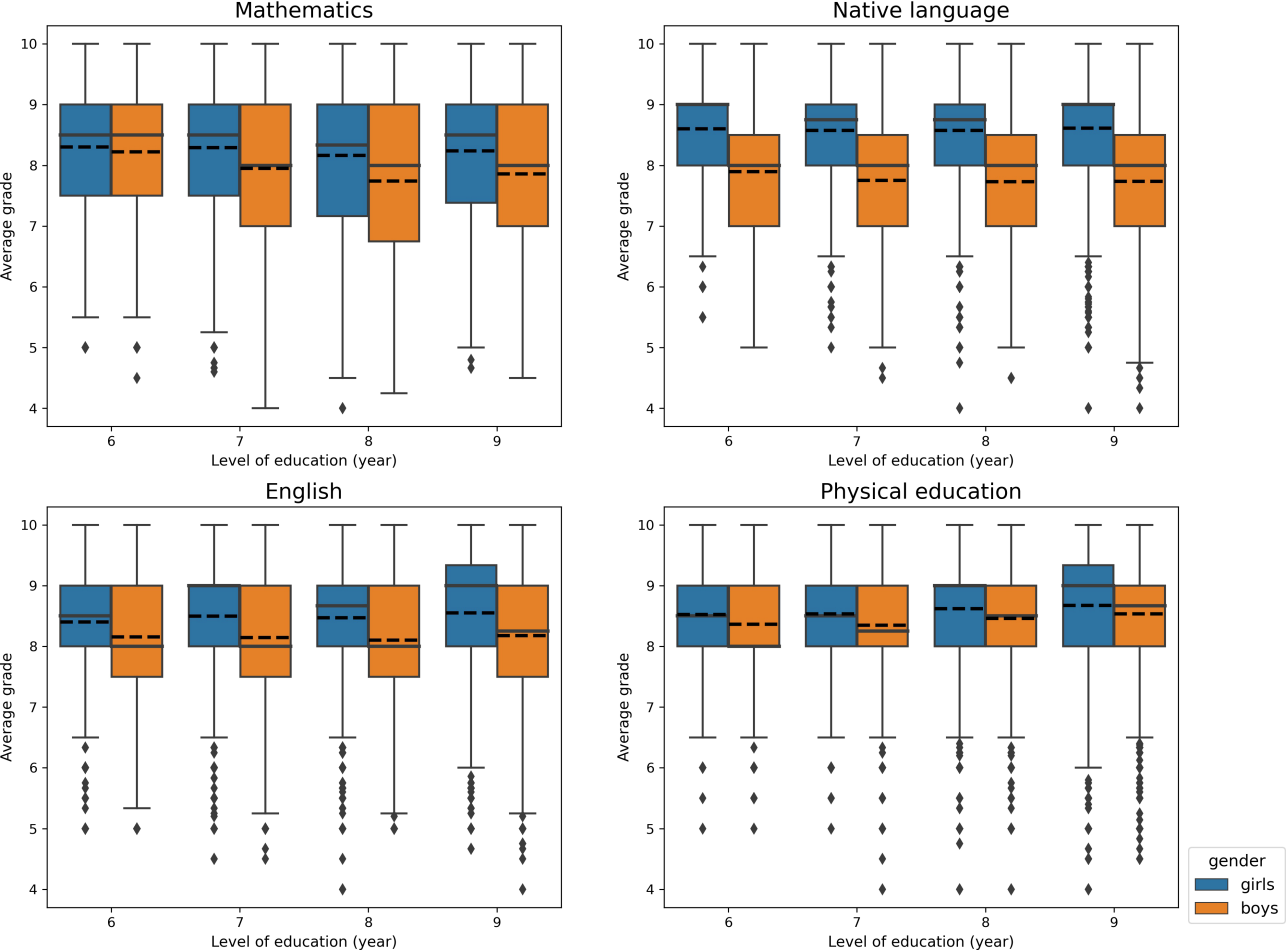
Boxplot of school performance in years 6,7,8 and 9 separately for girls and boys. The dotted line shows the mean and the solid line the median. Girls outperformed boys in all years and subjects statistically significantly (p<0.001).

Boys and girls performed differently in school years 6 to 9 (**Figure 3**). In year 6, the difference in academic performance was apparent in mathematics, native language, and English and the difference increased during middle school (years 7 through 9) in all three academic subjects. The sex difference in longitudinal changes in performance through years 6 to 9 was evident in all academic subjects (**eTable7** and **eTable8** in the Supplement), and in year 9 the gap between sexes was largest in native language (0.88 grade points) (**eTable8**). In physical education, girls performed better in year 6 but the sex difference decreased during later school years (**Figure 3**, **eTable7**, **eTable8**).

## Discussion

This large population-based study utilized the unique Finnish registers to estimate the timing of puberty and its relationship with school performance during adolescence. In both sexes, age at PHV was associated with the grades in physical education, and in girls, with performance in mathematics and English. Age at PHV neither explained the differences in academic performance in boys nor the dramatic decrease in boys’ grades in mathematics after the transfer from elementary school to middle school.

The mean ages at PHV estimates (13.54 years in boys, and 11.43 years in girls) were in agreement with the recent report from Sweden of children born in 1974 (13.66-13.73 years in boys and 11.83-12.02 years in girls) (37), especially after correction for a putative secular trend toward earlier pubertal timing for both sexes. Similarly, Ohlsson et al. reported recently that age at PHV in boys born in 1996 was 13.70 years (14). Thus, our estimates for the age at PHV in both sexes appear reliable. In girls, age at PHV was positively associated with grades in mathematics across the study years yet the effect sizes being relatively small. In boys, however, age at PHV showed no association with academic performance. Previous findings in girls have been contradictory (15, 38, 39). In a recent study of 13,477 British children, however, the General Certificate of Secondary Education (GSCE) score and age at menarche in girls correlated (18), which also suggests a beneficial effect of later development on academic performance. Direct comparison between these studies is unfortunately inappropriate given the apparent differences in study methodology. In contrast, pubertal timing and English grades in year 9 showed an inverse association among girls. Although speculative, this may reflect neuromodulatory effects of estrogens on the brain, given that estrogen receptors are densely expressed in brain regions that are important for cognitive functions, and in women, the activity of these regions and performance in cognitive tasks appear to be modulated by estrogen (40–44). In particular, performance in some verbal tasks and estrogen levels change in parallel in women during the menstrual cycle (45). On the other hand, adolescents are now constantly exposed to the English language through media and digital/electronic games, which may modify their interest in formal language learning.

The decrease in boys’ grades in mathematics and overall weaker performance in academic subjects in middle school was unexplained by age at PHV. The grades in mathematics among boys decreased across the transfer from elementary school to middle school and this phenomenon also occurred in the comprehensive schools that had both elementary and middle schools physically in the same building. The educational system changes, however, in the transfer from school year 6 to 7 from one classroom teacher to a subject teacher-based system. The gap between sexes was independent of the main language of the school (Finnish or Swedish). Due to similar school systems in other countries, this topic warrants further investigation. The weaker school performance of boys in Finland has been reported in previous studies (21, 36), and the proposed reasons include differences in motivation and attitudes towards learning, bullying, time spent on tasks, and expectations based on gender (36, 46). According to a recent report from Finland, boys’ interest in education decreased gradually and in year 8 girls considered all subjects more important than boys (46). Boys were bullied more (47) and exhibited a lower sense of belonging to the school, and these experiences were associated with weaker school performance (46). The Finnish National Agency for Education has made a guide to promote equality in comprehensive school and the impact of the new curriculum remains to be seen.

In both sexes, age at PHV was positively associated with the grades in physical education. This might reflect a puberty-related change in physical activity, yet a consistent relationship between the two has not been reported (48–57). A recent Slovenian study reported that physical activity declined with age but was not explained by the timing of the growth spurt (58). As discussed in several of the above studies, the changes in body composition during adolescence and physical self-perception are major correlates of physical activity in girls (54, 57) and in our study the effect sizes were higher in girls than in boys. It also seems plausible that earlier developers are clumsier and thereby give their peers an advantage in physical education classes. Indeed, the onset of the growth spurt in both sexes has been observed to be associated with impaired coordination (59) and a negative relationship between motor competence and being in growth spurt has been reported (60). Finally, it is possible that the observed relationship between the timing of puberty and grades in physical education is confounded by the lack of interest in physical exercise in the school setting. Given that earlier pubertal timing is related to higher risks for angina, hypertension, and type two diabetes (61), the relationship between lower grades in physical education and early pubertal maturation calls for adaptation of the school system, *i.e.* low physical education grades may not act as motivators for a life-long exercise habit so urgently needed in the modern Western society fighting against obesity and related co-morbidities.

The strengths of this study include the population-based setting, large study cohort, longitudinal data collection and design, and collection of growth and school performance data from official registries instead of surveys. Regarding limitations, no data was available on when the children started school, and, thus, a small fraction of the children may have been in a different year level from what we assumed based on their date of birth. In addition, data on final heights were not available. Children born outside of Finland were excluded from the analyses and, consequently, the effects of increased immigration were not assessed (62, 63). In Finland, children are generally assigned to their local middle school, there are no school fees, and secondary applicants are rare. This may create regional differences in academic performance between school districts. Nevertheless, the socio-economic status in the school catchment area showed no association with school performance in year 9. In the 2018 PISA survey, family-level socio-economic status was associated with reading proficiency as well as performance in mathematics and science (21). Unfortunately, we had no data on socio-economic status on a family level. It is possible that the area-level socioeconomic data may not fully capture the impact of socioeconomic factors on academic achievement and therefore the lack of family-level socioeconomic data is a limitation of the current study. Nevertheless, in the city of Espoo the lack of expensive private schools and greater funding for schools located in more disadvantaged areas (64) may have plateaued the effect of socioeconomic status. Can the present results be applied to different countries? Given that education systems fairly similar to that in Finland are in use in several other European countries and in Northern America, the generalizability of our results should be good (65, 66).

To the best of our knowledge, this is the first study to evaluate the relationship between the timing of puberty and academic performance in a representative population-based longitudinal cohort of Generation Z. The timing of puberty was associated with academic achievement only in girls, and boys’ weaker performance in academic subjects in middle school was unexplained by the timing of puberty. Due to the tremendous effect of education on later health and wellbeing, every effort should be taken to nullify the sex difference in school performance and to provide equal educational opportunities for all.

## Data availability statement

The data that support the findings of this study are available from Helsinki University Hospital and the city of Espoo. Restrictions apply to the availability of these data, which were used under license for this study. Data are available from the authors with the permission of Helsinki University Hospital and the city of Espoo. Requests to access the datasets should be directed to Taneli Raivio, taneli.raivio@helsinki.fi.

## Ethics statement

Ethical review and approval was not required for the study on human participants in accordance with the local legislation and institutional requirements. Written informed consent from the participants’ legal guardian/next of kin was not required to participate in this study in accordance with the national legislation and the institutional requirements.

## Author contributions

TR conceptualized, designed, and supervised the study, collected and interpreted data, and drafted, reviewed and revised the manuscript. MS collected, analyzed and interpreted the data, carried out statistical analyses, and drafted, reviewed and revised the manuscript. MH conceptualized, designed, and supervised the study, and drafted, reviewed and revised the manuscript. SK conceptualized and designed the study, and drafted, reviewed and revised the manuscript. PM drafted, reviewed and revised the manuscript. TV reviewed and revised the manuscript. OR designed the study and reviewed and revised the manuscript. AT collected data and provided technical and material support. All authors approved the final manuscript as submitted and agree to be accountable for the content of the work.

## Funding

This project was funded by the Foundation for Pediatric Research, Emil Aaltonen foundation and the Research Funds of the Helsinki University Hospital. The funders had no role in the design and conduct of the study.

## Conflict of interest

The authors declare that the research was conducted in the absence of any commercial or financial relationships that could be construed as a potential conflict of interest.

## Publisher’s note

All claims expressed in this article are solely those of the authors and do not necessarily represent those of their affiliated organizations, or those of the publisher, the editors and the reviewers. Any product that may be evaluated in this article, or claim that may be made by its manufacturer, is not guaranteed or endorsed by the publisher.
