# Supplementary material for: Timing of puberty and school performance: A population-based study
Source: Front Endocrinol (Lausanne). 2022 Aug 5;13:936005. doi: 10.3389/fendo.2022.936005 (PMC9388756; doi:10.3389/fendo.2022.936005)
Supplement: Supplementary file 1 [file DataSheet_1.docx]

**Supplementary Materials**

**eMethods**

**eFigure1**

**eFigure2**

**eTable1**

**eTable2**

**eTable3**

**eTable4**

**eTable5**

**eTable6**

**eTable7**

**eTable8**

**eMethods**

**Modelling of growth and determination of age at peak height velocity**

Python programming language (Python 3.7.4) was used for all calculations to determine age at peak height velocity (PHV). In the first method, a 7^th^ degree polynomial function was fitted to the growth data and the best fit was chosen using least-squares with NumPy library polyfit-function. A 7^th^ degree polynomial was used as a very small degree polynomial function leads to smoothening and possibly misses information, whereas a large degree polynomial function is expected to lead to overfitting. The polynomial function for the growth in height was derived with polyder-function to obtain the growth rate function. The zero points for the growth rate function’s derivatives, representing the stationary points for growth, were determined and the local maximum with the highest growth rate between 7.5-17.5 years of age was chosen to represent the age at PHV. eFigure 1 represents an example of the measuring points (red), the growth function (blue) and the growth rate function (yellow).


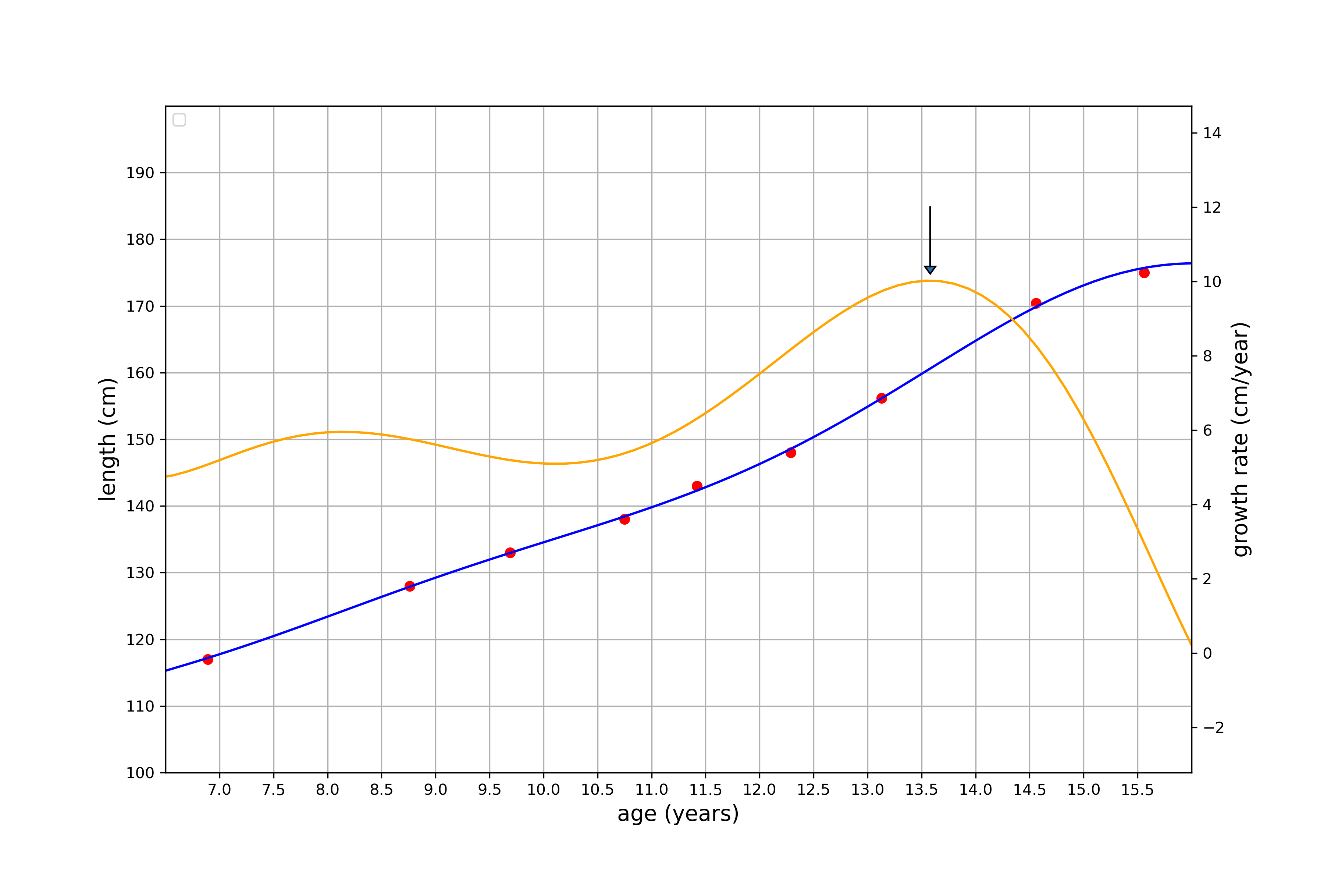


**eFigure 1 The growth function (blue) fitted with 7th degree polynomial to the measuring points (red). The age at PHV is 13.58 years (arrow).**

In the second method, a polynomial between 6 and 9 degrees was chosen for each person individually using a cross-validation technique with the Scikit-learn library to avoid over- or underfitting. In this case, leave-one-out cross-validation was used (*i.e.* one measuring point was left out at a time, in our case six times a different measuring point). The fit was compared with the measuring points by counting RMSE-value for each of the six cases. For all polynomials, ranging from 6 to 9 degrees, the average of the six RMSE-values were determined and the polynomial with the smallest average value was chosen.

**Conditions for growth data**

The condition of six or more height measurements was not fulfilled in 7951 children, and they were omitted from analyses. In addition to the condition of at least six measuring points, the following conditions were applied to ascertain sufficiency of data for reliable determination of age at PHV. Some of the conditions were separate for boys and girls due to differences in growth patterns.

For boys, the following conditions were applied:

1. height measurements up to the age of 13 years, as boys reach PHV at an average age of 13.5-14 years (1,2). This led to omission of 599 boys.
2. at least two height measurements between ages 10 and 15 years: one between ages 10 and 12.5 years and one between 12.5 and 15 years, and the difference between these measuring points could be no more than 2.3 years. This led to omission of 439 boys.
3. if the last height measure was before the age of 14.5 years, and the last height measurement was under 160 cm, the growth curves were manually checked to ensure that a correct age at PHV was chosen. In some cases this led to omission because no growth spurt was evident. This led to omission of 45 boys.

For girls, the following conditions were applied:

1. height measurements at least up to the age of 11 years, because girls reach PHV at an average age of 11.5-12 years (2,3). This led to omission of 244 girls.
2. at least two height measurements between ages 8 and 13 years: one between ages 8 and 10.5 years, and one between 10.5 and 13 years, and the difference between these measures could be no more than 2.3 years. This led to omission of 1044 girls.
3. If the last height measure was before the age of 12.5 years, and the last height measurement was under 150 cm, the growth curves were checked manually. In some cases this led to omission because no growth spurt was evident. This led to omission of 64 girls.

The following conditions were applied for both sexes:

1. The PHV had to be over 4 cm/year.
2. At least one height measurement was required within one year of the age at PHV.
3. Age at PHV had to be at least one year after the first measuring point
4. Age at PHV had to be prior to the last measuring point
5. If two possible local maximums for age at PHV were detected, the charts were manually checked

The additional conditions (listed above) were unmet in 3524 children with the 7^th^ degree polynomial function and in 3579 children with the chosen degree polynomial function. In the final dataset, age at PHV was determined with the 7^th^ degree polynomial function for 14,158 children (of which 13,285 were born in Finland) and with the chosen polynomial function for 14,103 children.

**Validation of the method**

The two different methods were validated using a random test set of 60 children, 30 boys and 30 girls. Three experienced pediatric endocrinologists independently checked the growth data of these cases and manually picked the point that represented the age at PHV. These manual results were compared with the results from the polynomial modelling. Only 59 cases were compared because in one case the age at PHV was undefinable manually due to steady growth. In this validation set, the measuring points began at an average age of 5.88 years and ended at the age of 16.75 years. These cases had on average 11.51 measuring points and therefore the distance between measuring points was approximately one year.

eTable1 summarizes the results from the comparison. The Pearson correlation coefficient was slightly better for the 7^th^ degree polynomial than for the polynomial chosen individually. In analyzes 7^th^ degree polynomial was used though age at PHV was determined with both methods.

The mean of all residuals of all individually fitted polynomial functions (7th degree polynomial function) were counted. The mean was 0 and SD was 0.54. The distribution of all residuals of all individual growth curves is shown in eFigure2. The steepness of the distribution varies from person to person which explains the peak in the figure but generally the difference between the expected value and observed value is less than 2 centimeters. eTable5 summarizes other characteristics of the data.


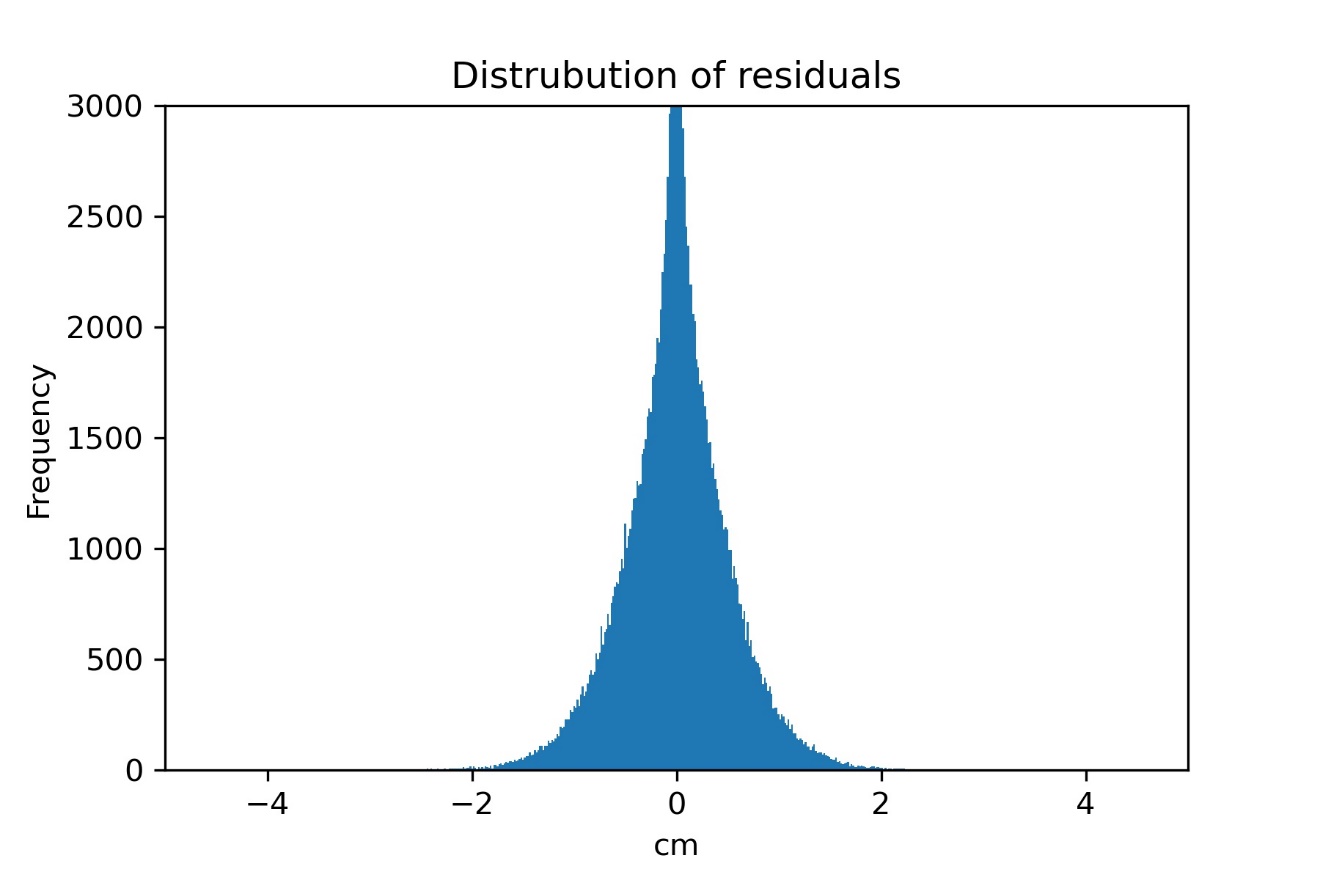


**eFigure 2 The distribution of all residuals of all individual growth curves**

**eTable 1 The age at PHVs from different polynomial functions were compared with results from three experienced pediatric endocrinologists who manually picked the point that represented the age at PHV.**

|  |  | **ENDO 1** | **ENDO 2** | **ENDO 3** | **chosen degree of 6-9** |
| --- | --- | --- | --- | --- | --- |
| **7^th^ degree** | **Pearson correlation coefficient (p-value)** | 0.948 (<0.001) | 0.939 (<0.001) | 0.935 (<0.001) | 0.975 |
|  | **R2** | 0.896 | 0.882 | 0.880 | 0.952 |
|  | **Adjusted R2** | 0.894 | 0.880 | 0.878 | 0.951 |
|  | **Average difference in years (SD)** | 0.072 (0.441) | 0.083 (0.464) | 0.038 (0.459) | 0.060 (0.304) |
| **chosen degree of 6-9** | **Pearson correlation coefficient (p-value)** | 0.928 (<0.001) | 0.924 (<0.001) | 0.922 (<0.001) |  |
|  | **R2** | 0.861 | 0.855 | 0.850 |  |
|  | **Adjusted R2** | 0.859 | 0.852 | 0.847 |  |
|  | **Average difference in years (SD)** | 0.012 (0.532) | 0.023 (0.541) | -0.022 (0.537) |  |
| **4^th^ degree** | **Pearson correlation coefficient** | 0.600 | 0.616 | 0.619 |  |
| **5^th^ degree** | **Pearson correlation coefficient** | 0.802 | 0.805 | 0.819 |  |
| **6^th^ degree** | **Pearson correlation coefficient** | 0.923 | 0.918 | 0.922 |  |
| **8^th^ degree** | **Pearson correlation coefficient** | 0.935 | 0.938 | 0.926 |  |

ENDO represents different pediatric endocrinologists, and the degrees indicate different polynomial functions used for comparison. The average difference in the age at PHV derived from the 7^th^ degree polynomial function and pediatric endocrinologists was 0.038-0.083 years (14-30 days) with a standard deviation of 0.441-0.464 years depending on the endocrinologist. The average difference between the result with chosen polynomial of degree 6-9 and the pediatric endocrinologists was -0.022-0.023 years (8-9 days) with a SD of 0.532-0.541 years depending on the endocrinologist. The Pearson correlation coefficient was slightly better for the 7^th^ degree polynomial than for the polynomial chosen individually. The 7^th^ degree polynomial and polynomial of chosen degree correlated well. There were also differences between the endocrinologists with the largest difference of 1.6 years. The table further shows the correlation coefficients for 4^th^, 5^th^, 6^th^ and 8^th^ degree polynomial. For small degree polynomials the correlation coefficient was clearly lower than for 7^th^ degree, but for 6^th^ and 8^th^ degree polynomials almost the same as for 7^th^ degree. Very high degree polynomials however lead to multi-peak curves and overfitting. The chosen degree polynomial chose most often 6^th^ or 7^th^ degree polynomial.

**eTable 2 The structure of the linear mixed model***.*

| Level | Variables |
| --- | --- |
| Residential area (n=18–21) | income level in the residential area, education level in the residential area |
| School (n=29–31 for middle schools and n=55-57 for elementary schools) |  |
| Child (n=4392- 6055 depending on sex, school year and subject) | age at PHV, month of birth |
|  | dependent variable: average grade in mathematics, native language, English or physical education |

**eTable3 Linear mixed model comparison with maximum likelihood in year 9: the null model and the final model. According to the Akaike Information Criterion and p-values, in girls the final model described better the correlations in mathematics, English and physical education and in boys in mathematics, native language and physical education. We accepted**

**p-value<0.01 to indicate statistical significance.**

|  |  | Mathematics | | Native language | | English | | Physical education | |
| --- | --- | --- | --- | --- | --- | --- | --- | --- | --- |
|  |  | **Null** | **Final**  **β (95% CI) p-value** | **Null** | **Final**  **β (95% CI) p-value** | **Null** | **Final**  **β (95% CI) p-value** | **Null** | **Final**  **β (95% CI) p-value** |
| Girls | **Residential area-level variables** | | | | | | | | |
|  | **income level** |  | 8e-06 (-2e-05, 3e-05)  p= 0.53 |  | -1e-07 (-2e-05, 2e-05) p= 0.99 |  | -4e-06 (-4e-05, 3e-05) p= 0.81 |  | -6e-06 (-3e-05, 2e-05) p= 0.63 |
|  | **education level** |  | 0.0070 (-0.025, 0.040) p= 0.66 |  | 0.0196 (-0.009, 0.048) p= 0.17 |  | 0.0244 (-0.015, 0.064) p= 0.22 |  | 0.0273 (-0.003, 0.058) p= 0.08 |
|  | **Child-level variables** | | | | | | | | |
|  | **age at PHV** |  | **0.0434 (0.015, 0.072) p=** **0.003** |  | 0.0054 (-0.017, 0.027) p= 0.63 |  | **-0.0468(-0.072, -0.021) p<0.001** |  | **0.1059 (0.084, 0.128) p<0.001** |
|  | **month of birth** |  | -0.0061 (-0.016, 0.004) p= 0.23 |  | -0.0057 (-0.013, 0.002) p= 0.13 |  | -0.0032 (-0.012, 0.006) p= 0.48 |  | -0.0062 (-0.014, 0.002) p= 0.11 |
|  | **ICC residential area** | 0.035 | 0.028 | 0.025 | 0.012 | 0.055 | 0.044 | 0.008 | 0.000 |
|  | **ICC school within residential area** | 0.036 | 0.034 | 0.094 | 0.085 | 0.075 | 0.072 | 0.138 | 0.114 |
|  | **AIC** | 17581.4 | **17576.4** | 13820.6 | 13821.2 | 15383.1 | **15374.5** | 14468.3 | **14382.2** |
| Boys | **Residential area -level variables** | | | | | | | | |
|  | **income level** |  | 5e-06 (-2e-05, 3e-05) p=0.68 |  | 4e-06 (-2e-05, 3e-05) p=0.67 |  | -2e-06 (-3e-05, 3e-05) p=0.88 |  | -6e-06 (-2e-05, 1e-05) p=0.51 |
|  | **education level** |  | 0.0151 (-0.017, 0.047) p=0.34 |  | 0.0120 (-0.013, 0.036) p=0.33 |  | 0.0158 (-0.020, 0.052) p=0.38 |  | 0.0186 (-0.004, 0.040) p=0.10 |
|  | **Child-level variables** | | | | | | | | |
|  | **age at PHV** |  | -0.0002 (-0.030, 0.030) p=0.99 |  | 0.0017 (-0.022, 0.025) p=0.89 |  | -0.0281 (-0.054, 0.003) p=0.03 |  | 0.0258 (0.005, 0.047) p=0.01 |
|  | **month of birth** |  | -0.0123 (-0.022, -0.003) p=0.01 |  | -0.0082 (-0.016, 0.001) p=0.04 |  | -0.0032 (-0.012, 0.005) p=0.46 |  | **-0.0172 (-0.024, -0.010) p<0.001** |
|  | **ICC residential area** | 0.044 | 0.033 | 0.000 | 0.000 | 0.068 | 0.062 | 0.009 | 0.002 |
|  | **ICC school within residential area** | 0.017 | 0.016 | 0.081 | 0.066 | 0.027 | 0.027 | 0.066 | 0.061 |
|  | **AIC** | 20533.6 | **20531.0** | 16763.9 | **16762.9** | 17321.6 | 17322.9 | 15959.3 | **15932.2** |

AIC= Akaike Information Criterion, ICC= Intraclass correlation coefficients, CI=confidence interval, PHV=peak height velocity

**eTable 4 Means and standard deviations of school performance in girls and boys in school year 9. The excluded group consists of those who had data on school performance but for whom determination of age at PHV was not possible.**

|  | **Boys** | | **Girls** | |
| --- | --- | --- | --- | --- |
|  | **Included** | **Excluded** | **Included** | **Excluded** |
| **Mathematics year 9**  **mean (SD)** | 7.86 (1.35) | 7.53 (1.38) | 8.24 (1.27) | 7.87 (1.33) |
| **Native language year 9 mean (SD)** | 7.74 (1.06) | 7.43 (1.10) | 8.61 (0.96) | 8.39 (1.05) |
| **English year 9**  **mean (SD)** | 8.18 (1.13) | 7.88 (1.26) | 8.55 (1.12) | 8.24 (1.26) |
| **Physical education year 9**  **mean (SD)** | 8.53 (0.94) | 8.29 (1.02) | 8.67 (0.99) | 8.39 (1.07) |
| **Income level**  **median (SD)** | 40,806 (8565 €) | 40,372 € (8464€) | 41,240 € (9078 €) | 40,304 € (8356 €) |
| **Education level**  **mean (SD)** | 47.40 % (7.45 %) | 47.07 % (7.15 %) | 47.77 % (7.54 %) | 47.10 % (7.21 %) |

**eTable 5 Characteristics for the data (when measuring points start, end and how many of them) and the characteristics for age at PHV with the 7^th^ degree polynomial**

|  | **Average** |
| --- | --- |
| **Measuring points start** | 4.17 years (±2.15 SD) |
| **Measuring points end** | 16.59 years (±1.24SD) |
| **Measuring points in total** | 13.31 (±3.72 SD) |
| **Mean (SD) age at PHV** | \| girls \| 11.43 years (±1.18 SD) \| \| --- \| --- \| \| boys \| 13.54 years (±1.17 SD) \| |
| **Percentiles for age at PHV** | \| girls \| 25%: 10.68 years  50%: 11.44 years  75%: 12.17 years \| \| --- \| --- \| \| boys \| 25%: 12.79 years  50%: 13.55 years  75%: 14.31 years \| |

**eTable 6 The correlations between month of birth and school performance calculated using linear mixed model.**

|  | | **Mathematics** | | | **Native language** | | | **English** | | | **Physical education** | | |
| --- | --- | --- | --- | --- | --- | --- | --- | --- | --- | --- | --- | --- | --- |
|  |  | **n** | **Coef for month of birth**  **(95% CI)** | **p-value** | **n** | **Coef for month of birth**  **(95% CI)** | **p-value** | **n** | **Coef for month of birth**  **(95% CI)** | **p-value** | **n** | **Coef for month of birth**  **(95% CI)** | **p-value** |
| **Girls** | **Year 9** | | | | | | | | | | | | |
|  |  | 5366 | -0.0061 (-0.016, 0.004) | 0.23 | 5152 | -0.0057 (-0.013, 0.002) | 0.14 | 5141 | -0.0032 (-0.012, 0.006) | 0.48 | 5252 | -0.0062 (-0.014, 0.001) | 0.11 |
|  | **Year 7** | | | | | | | | | | | | |
|  |  | **5241** | **-0.0157 (-0.025, -0.007)** | **<0.001** | **4997** | **-0.0097(-0.016,-0.003)** | **0.004** | 4937 | -0.0105(-0.019,-0.002) | 0.01 | **5107** | **-0.0113(-0.018,-0.005)** | **<0.001** |
|  | **Year 6** | | | | | | | | | | | | |
|  |  | **4540** | **-0.0281 (-0.037, -0.019)** | **<0.001** | **4338** | **-0.0201(-0.027,-0.013)** | **<0.001** | **4392** | **-0.0179(-0.027,-0.009)** | **<0.001** | **4441** | **-0.0201(-0.026,-0.014)** | **<0.001** |
| **Boys** | **Year 9** | | | | | | | | | | | | |
|  |  | 6055 | -0.0123 (-0.022, -0.003) | 0.01 | 5807 | -0.0082 (0.016, 0.000) | 0.04 | 5716 | -0.0032 (-0.012, 0.005) | 0.46 | **5985** | **-0.0172(-0.024,-0.010)** | **<0.001** |
|  | **Year 7**  **Year 7** | | | | | | | | | | | | |
|  |  | **5917** | **-0.0154 (-0.024, -0.007)** | **<0.001** | **5673** | **-0.0093(-0.016,-0.002)** | **0.009** | 5462 | -0.0107(-0.019,-0.003) | 0.01 | **5826** | **-0.0194(-0.025,-0.013)** | **<0.001** |
|  | **Year 6** | | | | | | | | | | | | |
|  |  | **5102** | **-0.0181 (-0.027, -0.009)** | **<0.001** | **4894** | **-0.0135(-0.021,-0.006)** | **<0.001** | **4856** | **-0.0155(-0.024,-0.007)** | **<0.001** | **5040** | **-0.0151(-0.021,-0.009)** | **<0.001** |

**eTable 7 Change in school performance during progression from year 6 to 9 calculated using linear regression (average grade as dependent variable and school year as independent variable). The p-value for differences between sexes was calculated with multiple linear regression, sex as a categorical variable.**

|  | **Girls** | | **Boys** | | **p-value** |
| --- | --- | --- | --- | --- | --- |
|  | **β (95% CI)** | **p-value** | **β (95% CI)** | **p-value** |  |
| **Mathematics** | **-0.0325 (-0.047, -0.018)** | **<0.001** | **-0.1276 (-0.142, -0.113)** | **<0.001** | **<0.001** |
| **Native language** | 0.0040 (-0.007, 0.015) | 0.47 | **-0.0497 (-0.061, -0.038)** | **<0.001** | **<0.001** |
| **English** | **0.0424 (0.029, 0.056)** | **<0.001** | 0.0027 (-0.010, 0.015) | 0.68 | **<0.001** |
| **Physical education** | **0.0540 (0.043, 0.065)** | **<0.001** | **0.0631 (0.053, 0.073)** | **<0.001** | **<0.001** |

**eTable** **8 Means and standard deviations in school performance in girls and boys in school years 6, 7 and 9. The differences between sexes are statistically significant in all subjects (p<0.001).**

|  | **Year 6** | |  | | **Year 7** | |  | | **Year 9** | |  | |
| --- | --- | --- | --- | --- | --- | --- | --- | --- | --- | --- | --- | --- |
|  | **Girls**  **Mean (SD)** | **Boys**  **Mean (SD)** | | **difference** | **Girls**  **Mean (SD)** | **Boys**  **Mean (SD)** | | **difference** | **Girls**  **Mean (SD)** | **Boys**  **Mean (SD)** | | **difference** |
| **Mathematics** | 8.30 (1.07) | 8.22 (1.10) | | 0.08 | 8.29 (1.14) | 7.95 (1.18) | | 0.35 | 8.24 (1.27) | 7.86 (1.35) | | 0.38 |
| **Native language** | 8.60 (0.83) | 7.90 (0.91) | | 0.71 | 8.57 (0.83) | 7.75 (0.93) | | 0.82 | 8.61 (0.96) | 7.74 (1.06) | | 0.88 |
| **English** | 8.40 (1.05) | 8.16 (1.05) | | 0.25 | 8.49 (1.07) | 8.14 (1.06) | | 0.35 | 8.55 (1.12) | 8.18 (1.13) | | 0.37 |
| **Physical education** | 8.52 (0.75) | 8.36 (0.81) | | 0.16 | 8.54 (0.78) | 8.35 (0.81) | | 0.19 | 8.67 (0.99) | 8.53 (0.94) | | 0.14 |

**References**

1. Ohlsson C, Bygdell M, Celind J, et al. Secular Trends in Pubertal Growth Acceleration in Swedish Boys Born From 1947 to 1996. *JAMA Pediatr*. 2019;173(9):860-865. doi:10.1001/jamapediatrics.2019.2315

2. Holmgren A, Niklasson A, Gelander L, Aronson AS, Nierop AFM, Albertsson-Wikland K. Insight into human pubertal growth by applying the QEPS growth model. *BMC Pediatr*. 2017;17:107. doi:10.1186/s12887-017-0857-1

3. Aksglaede L, Olsen LW, Sørensen TIA, Juul A. Forty Years Trends in Timing of Pubertal Growth Spurt in 157,000 Danish School Children. *PLoS One*. 2008;3(7):e2728. doi:10.1371/journal.pone.0002728
